# Vitamin D and Inhibition of Melanogenesis: Examination of Liposomal Vitamin D Nanocarrier Anti‐Melanogenesis Activity on B16 F10 Cell Line

**DOI:** 10.1002/fsn3.70302

**Published:** 2025-05-19

**Authors:** Azita Bahrami, Alireza Farasat, Leila Zolghadr, Yalda Sabaghi, Nematollah Gheibi

**Affiliations:** ^1^ Cellular and Molecular Research Center, Research Institute for Prevention of Non‐Communicable Diseases Qazvin University of Medical Sciences Qazvin Iran; ^2^ Monoclonal Antibody Research Center Avicenna Research Institute, ACECR Tehran Iran; ^3^ Department of Chemistry, Faculty of Science Imam Khomeini International University Qazvin Iran

**Keywords:** apoptosis, atomic force microscope (AFM), B16 F10 cells, kinetic modeling, liposomes, vitamin D

## Abstract

Despite various treatment strategies employed thus far, malignant melanoma usually resists most of the interventions. The present work is an attempt to synthesize liposomes encapsulating vitamin D, evaluate their physicochemical properties, evaluate the release kinetics of vitamin D, and examine the cytotoxicity of both liposomal and free vitamin D on melanoma cells in vitro. The analysis revealed that the zero‐order model exhibited the best correlation (0.998) at acidic pH (5.5), indicating the most efficient vitamin D release from the nanoparticles. The results indicated that the IC50 values for cancer cells exposed to vitamin D‐containing liposomes were 35.08 and 28.96 μg/mL after 24–48 h. Compared to those treated with free vitamin D, flow cytometry results further demonstrated a higher apoptosis in B16 F10 cells exposed to vitamin D‐containing liposomes. Moreover, vitamin D‐containing liposomes induced the most significant nanomechanical alterations, with the most pronounced decrease in the expression of the PI3K/AKT1 gene observed in the liposomal vitamin D treatment group.

## Graphical abstract

Melanoma is indeed a serious form of skin cancer that can lead to death if not diagnosed and treated early. The liposomal vitamin D can be an effective agent for preventing and treating melanoma cells (B16 F10). Melanoma cells (B16 F10) were successfully induced to undergo apoptosis in vitro by liposomal vitamin D. AFM analyses showed that B16 F10 cells exposed to liposomal vitamin D had a high cell–cell adhesion force and elastic modulus.

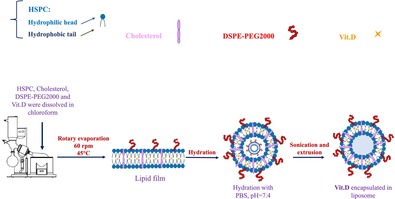

## 1 Introduction

Melanoma is a particularly aggressive skin cancer and, if not treated promptly, it can be lethal (Fan et al. 2023). Despite the availability of various treatment modalities, the outcomes for melanoma patients have often been disappointing, largely due to the ineffectiveness of current therapies (Rantala et al. 2022). One of the primary reasons for this ineffectiveness is the inherent resistance of melanoma cells to many conventional drugs (Fan et al. 2023). Presently, patients with melanoma are treated with standard therapeutic approaches widely adopted in clinical practice. However, these treatments are frequently hindered by significant challenges like malignant side effects, drug resistance, and potential toxicity, all of which limit their overall efficacy. Consequently, to enhance treatment response, mitigate drug resistance, and improve the prognosis and survival rates of melanoma patients, it is imperative to explore alternative or adjunctive therapies (Mirzavi et al. 2021). In recent decades, nanotechnology has emerged as a promising frontier for cancer treatment and diagnosis. Numerous nanoparticles are known as potential candidates as drug delivery systems, demonstrating enhanced therapeutic efficacy in various cases.

Among these, liposomes have garnered particular attention given their non‐toxicity, safety, and biocompatibility, making them ideal drug carriers (Gupta et al. 2017; Abdelaziz et al. 2018). Liposomes are vesicular systems at the nanoscale that offer advantages over other nanoparticles, which is mainly due to their natural origin. This intrinsic composition allows liposomes to accept several active pharmaceutical ingredients, both hydrophilic and hydrophobic, thereby providing a versatile platform for drug delivery. The structural adaptability of liposomes permits the modification of pharmacokinetic properties, resulting in improved therapeutic outcomes. Moreover, liposomes can be engineered to incorporate various agents, like gold nanoparticles for treatment and diagnosis purposes, and attenuated bacteria for vaccine development. Compared to other nanoparticle drug delivery systems, liposomes are highly biocompatible and offer numerous benefits. They are capable of preventing drug degradation, allowing for size customization, facilitating targeted cell delivery, and enabling easy modification of surface properties (Zylberberg and Matosevic 2016; Antimisiaris et al. 2021). These attributes underscore the potential of nanoparticles as superior carriers in the quest to develop more effective and safer treatments for melanoma.

Because liposomes are mostly made of cholesterol and phospholipids, the building elements of biological membranes, they are biodegradable, biocompatible, and have minimal toxicity. These properties confer biological advantages. Peritoneal macrophages readily swallow liposomes with lipids such as phosphatidic and phosphatidylserine acid, although they are less likely to do so for neutral liposomes composed of positively charged liposomes containing stearyl amine and egg phosphatidylcholine. The liposome structure contains 30% mol% cholesterol, improves stability by reducing membrane permeability, and is employed in the manufacture of chemotherapeutic drugs. Liposomes, however, have difficulties due to their quick removal by reticuloendothelial systems. It is possible to conjugate polyethylene glycol (PEG) with the liposome membrane to stop absorption and degradation. Surface charge affects liposomes’ interaction with the mononuclear phagocytic system (Suk et al. 2016; Sabaghi et al. 2024).

As a prohormone, Vitamin D is synthesized in keratinocytes by converting 7‐dehydrocholesterol when exposed to UV light in the 290–320 nm wavelength (Tang et al. 2012). Vitamin D plays several critical roles, such as maintaining calcium homeostasis and exhibiting pleiotropic effects like regulating differentiation, proliferation, cell cycle, and apoptosis (Umar et al. 2018; Bikle 2004; Kubis and Piwowar 2015). In vitro studies have highlighted the inhibitory effects of active vitamin D on malignant melanoma cell lines via various antitumor mechanisms, like the regulation of differentiation and apoptosis (Piotrowska et al. 2019; Osborne and Hutchinson 2002). Studies have shown that vitamin D is needed for slowing tumor progression and disease dissemination (Slominski et al. 2018). Additional studies have indicated that levels of vitamin D decrease in the subjects and an inverse association exists between vitamin D levels and wound mitosis rate (Lim et al. 2018), tumor thickness (Wyatt et al. 2015; Newton‐Bishop et al. 2009), and histological type (Newton‐Bishop et al. 2015). Moreover, an inverse association exists between the concentration of serum vitamin D and mortality and melanoma, suggesting that a shortage of vitamin D is a significant risk factor for cancer (Yin et al. 2013; Bikle et al. 2016; Reichrath et al. 2016). Studies support the use of vitamin D in its active form for preventing and treating various diseases, like cancer (Ma et al. 2010; Slominski et al. 2017). Therefore, research provides substantial evidence for the potentials of vitamin D in melanoma treatment (Piotrowska et al. 2019; Osborne and Hutchinson 2002).

The principal goal of this word was to examine the effects of liposomal and free vitamin D on B16 F10 melanoma cells treatment. The toxicity effects of liposomal and free vitamin D on cancer cells were examined through the MTT assay. Through vitamin D encapsulation, we aimed to enhance its efficacy and delay degradation. The PI3K activation happens by growth factors binding to the receptors on the cell surface and initiation of the AKT pathway. Phosphorylated AKT can then trigger downstream targets like MAPK and mTOR, which are crucial for regulating survival and progression of cells. Previous studies have indicated that vitamin D is capable of downregulating the downstream effectors and the AKT pathway in melanoma cancer (Shariev et al. 2022). In Kizildag et al. (2010), the authors reported that using vitamin D to treat the K562 cell line resulted in decreased expression of mRNA of the BCL2 gene, while increasing apoptosis‐promoting gene expression such as BAX (Kizildag et al. 2010).

The effects of vitamin D on inducing apoptosis and comparing its impact in both free and liposomal forms were examined. The study examines the liposomes' physicochemical properties encompassing size, morphology, and zeta potential. Furthermore, the nanomechanical characteristics of the liposomes were investigated alongside the expression levels of BAX, BCL2, PI3K, and AKT1 genes. To achieve these aims, a variety of advanced techniques were utilized, including the MTT assay for cell viability, atomic force microscopy for detailed structural analysis, RT‐PCR for gene expression profiling, and flow cytometry for apoptosis quantification. Additionally, the study involved a critical evaluation of different types of kinetic models to determine the most effective model for the controlled vitamin D release.

## 2 Materials and Methods

### 2.1 Materials

The study used the B16 F10 cell line supplied by the Iranian Biological Resource Center. MTT and vitamin D were acquired from Sigma‐Aldrich, and additional consumables including chloroform were provided by Merck. Iran‐based BioIdea provided Trypsin–EDTA, the DMEM medium, fetal bovine serum (FBS), and penicillin and streptomycin. Avanti Polar Lipids provided DSPE‐PEG2000, HSPC, cholesterol, and a dialysis membrane, while Ebioscience's Annexin‐V Kit was used.

### 2.2 Synthesis of Liposomes

The synthesis of liposomes was carried out using a modified version of the standard thin film hydration technique (Molaveisi et al. 2021). Initially, a lipid mixture comprising hydrogenated soy phosphatidylcholine (HSPC), cholesterol, and 1,2‐distearoyl‐sn‐glycero‐3‐phosphoethanolamine‐N‐[methoxy (polyethylene glycol)‐2000] (DSPE‐PEG2000) was solved in ethanol and chloroform at a 3:7 (v/v) ratio. The molar ratios of the lipids were set at 70:25:5, respectively. 4.5 mg of vitamin D was added to this lipid solution in a round‐bottom flask. The solvent was evaporated at 49°C to achieve a thin lipid film. The resulting lipid film was thoroughly dried and subsequently rehydrated with 3 mL of phosphate‐buffered saline (PBS) at pH 7.4, manually shaken to ensure the film detached from the flask wall and dissolved uniformly. The rehydrated liposomal suspension was then homogenized at 3000 rpm for 5 min at 25°C to reduce the particle size. Following homogenization, the liposomes underwent probe sonication at 75% amplitude in an ice compartment for 10 min to further refine their size and uniformity. To ensure sterility and achieve the desired size distribution, using sterile mixed cellulose ester (MCE) filters (0.22 μm), the liposomal suspension was filtered seven times (Sigma‐Aldrich, USA). Finally, the liposomes were subjected to freeze‐drying in a lyophilizer for 48 h to obtain stable liposomal powders.

### 2.3 Determination of Encapsulation Efficiency (EE%)

Using ultracentrifugation, liposomes were isolated out of non‐entrapped vitamin D. To determine the volume of un‐entrapped vitamin D, a spectrophotometer (295 nm) was used. Using a standard curve, the vitamin D volume was evaluated (*R* ^2^ = 0.999).

**FIGURE 1:**
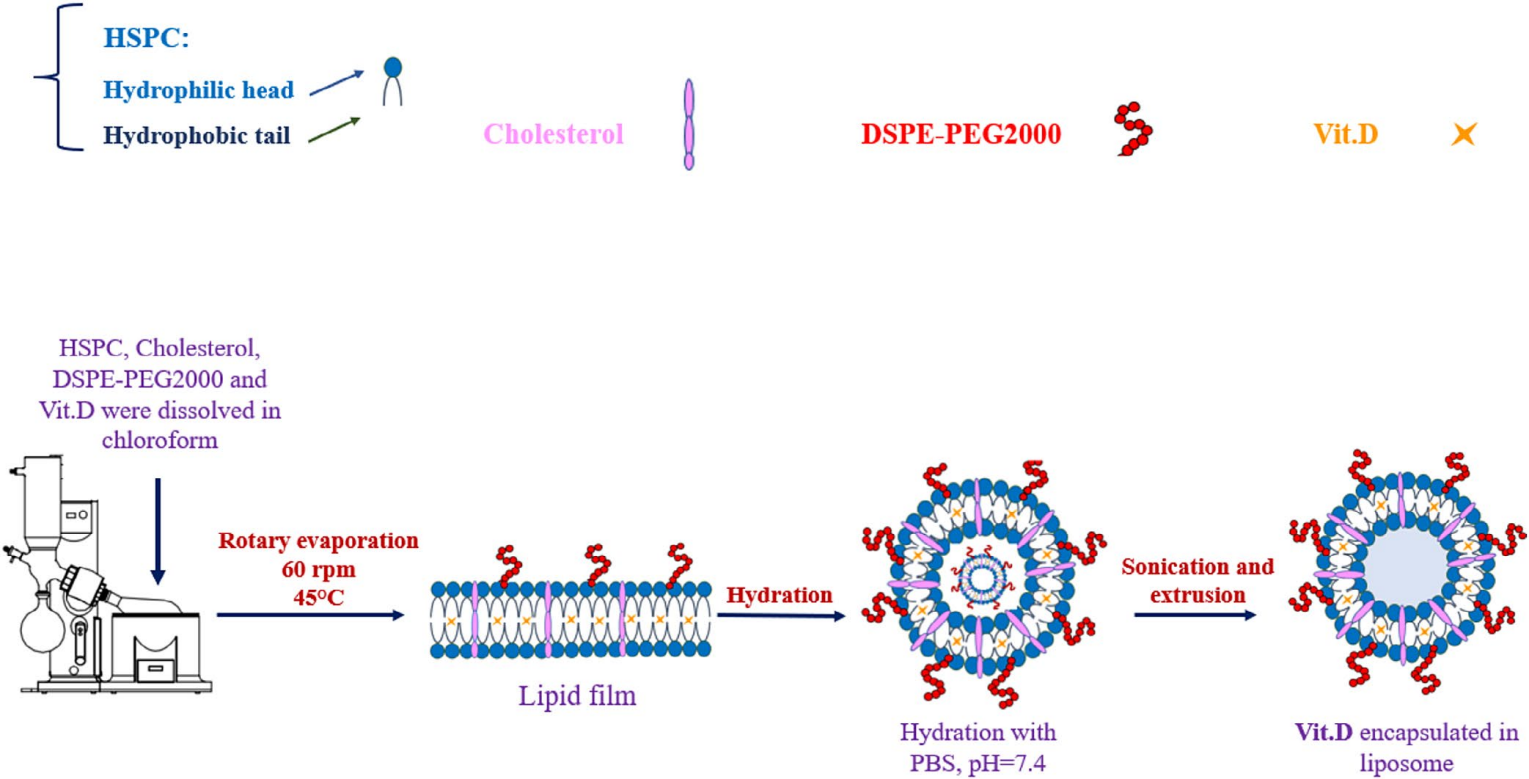
Preparation procedure of liposomes containing vitamin D.

Equation (1) yields the encapsulation efficiency (Brgles et al. 2008). The preparation of liposomes and vitamin D loading were carried out as summarized in Figure 1.

(1) $$\mathrm{EE}\%=\frac{\text{amount of vitamin}\;D\;\text{used}-\text{amount of free vitamin}\;D}{\text{amount of vitamin}\;D\;\text{used}}\times 100$$

### 2.4 Liposomes

#### 2.4.1 Particle Size and Zeta Potential

Light scattering with the NANO‐flex equipment was used to determine the nanoliposomes size (Particle Metrix, Germany). Diluting liposomes powder with PBS minimized light scattering effects, and particle size was measured using zeta mean diameter and polydispersity index. Using a ZETA‐check, the zeta potential of nanoparticles was determined at 41% moisture content and 25°C.

#### 2.4.2 FTIR

To determine the functional groups, FTIR (Bruker, USA) was used. The FTIR spectra analyses were done in 400–4000 cm^−1^ range.

#### 2.4.3 Morphology

The morphology of nanoliposomes was examined using SEM images. First, freeze‐dried liposomes were ground, and then imaging of the lyophilized samples was done through the SEM device (Hitachi).

### 2.5 Vitamin D Release Kinetics

A variety of models of kinetic are available for drug release characterization. Here, zero‐order, first‐order, Hixson‐Crowell, Higuchi, and Peppas were used for analyzing release kinetics. Drugs in the zero‐order model are released at a consistent pace, regardless of the concentration or quantity of drug. Equation (2) explains the drug release based on zero‐order. Where *Q*_0_ is the starting drug concentration in the solution, *Q*_t_ is the drug volume dissolved in time *t*, and *K*_0_ represents the zero‐order release constant given in concentration/time units. The drug's release following first‐order kinetics can be described using the Equation (3), where *Q*_t_ represents the initial amount of drug, *K* indicates the first‐order release constant, and *t* is time in hours. Higuchi introduced a model to explain drug release using Fickian diffusion principles. Equation (4) expresses the release of the drug based on this model. Based on Equation (5), Hixson‐Crowell illustrates release for different diameters and surface areas of particles. Where *k* refers to the Hixson‐Crowell constant and *Q*_t_ refers to the dosage released in time. Peppas et al. introduced a straightforward connection to picture drug release. The model is explained by Equation (6), which specifies the mechanism of release. Based on time, the drug release is represented by *M*_t_/*M*, the release exponent is *n*, and *K* represents the constant of the release rate (Dash et al. 2010; Paarakh et al. 2018).

(2) $$Q_{t}=Q_{0}+K_{0}t$$

(3) $$\log\;Q_{t}=\log\;Q_{0}+K_{0}t/2.303$$

(4) $$Q=k_{H}\;t^{½}$$

(5) $$Q_{t}=k_{t}^{n}$$

(6) $$M_{t}/M=K_{m}t^{n}$$

### 2.6 Cell Culture

The culture environment for B16 F10 cells included a temperature of 37°C, humidity of 95%, CO_2_ of 5%, and in DMEM complete culture medium containing 10% FBS and 1% penicillin, streptomycin. Using a phase contrast microscope (Italy) and a hemocytometer, the number of cells was counted at a density of 5 × 10^3^ cells per well. In 96‐well microplates, cells were plated in triplicate. Following a 24‐h period, B16 F10 cells viability was quantitatively analyzed. Two different forms of vitamin D (free and liposomal) with dosages of 10–150 μg/mL were given to the cells for 24 and 48 h.

### 2.7 Cell Viability Assay

The MTT test was used to assess liposomal and free vitamin D toxicity on B16 F10 melanoma cells. The test involved replacing treated cells' medium with a 100 μL/well MTT solution and incubating them at 37°C for 4 h. Then, the formazan salt was dissolved by extracting the MTT solution and adding 200 μL/well of DMSO. The cell viability was determined by a multi‐well plate reader (Bio Tek) and, as a reference wavelength, a formazan absorbance measurement at 570 nm.

### 2.8 Apoptosis Analysis

The analysis of treated cancer cells' apoptosis was done through flow cytometry. Apoptosis was assessed using a Bioscience kit and a flow cytometer (Dickinson, USA) with propidium iodide (PI) and Annexin V‐FITC labeling. The cell apoptosis kit's instructions were followed when seeding the cells in microplate wells with 0.3 × 10^6^ density. After that, for 24 and 48 h, they were given IC50 dosages of free vitamin D (50 and 40 μg/mL) and vitamin D‐containing liposomes (35 and 30 μg/mL) at 37°C. Harvested cells were rinsed by a PBS and then centrifuged for 5 min at 670 RCF. Then, 400 μL of PBS were used to resuspend the cell pellet. Subsequently, they were stained with 250 μL of Annexin V binding buffer. The cells were left in the dark for 15 min. Ultimately, a flow cytometer was utilized to assess the samples, and Flowjo software was employed to interpret the outcomes.

### 2.9 Analysis of the Cell Cycle

For 24–48 h, liposomal and free vitamin D were administered to B16 F10 cells in order to test the compounds' ability to cause cell cycle arrest. Following trypsin‐assisted cell harvesting, the pellet was fixed in 70% ethanol in PBS solution at −20°C for 1 h. Staining of fixed cells was done for 1 h at 37°C using 1 μL of PI (1 mg/mL) and 3 μL of RNase A (100 mg/mL) in PBS. FlowJo software was utilized to calculate cell percentages in the S, G0/G1, and G2/M phases of the cell cycle.

### 2.10 Atomic Force Microscopy (AFM)

After being on Petri dishes overnight, the cancer cells were treated with vitamin D‐containing liposomes and free form of vitamin D for 24 and 48 h. Samples were washed using PBS, fixed with a 0.5% glutaraldehyde solution, and dried. Using an AFM, the elasticity of the cells was examined. Ultrastructure and morphology were checked at 37°C, and image quality control was achieved. Following Equation (7), Young's modulus is as follows:

(7) $$F=\frac{2}{\pi }\tan\left(\alpha \right)\frac{E}{1-v^{2}}\delta^{2}$$

where *α* refers to half of a conical tip's opening angle *E* refers to Young's modulus, *F* represents loading force, *v* is Poisson's ratio at 0.5 suitable for cells, and *δ* indicates indentation (Pi et al. 2016).

### 2.11 RNA Isolation and cDNA Synthesis

B16 F10 cells were seeded in six‐well plates (25 × 10^4^) cells per well and incubated for 24 h at 37°C. Then, the cells were exposed to the IC50 dose of encapsulated and free vitamin D. Total RNA was determined after 24 and 48 h of incubation utilizing the Total RNA Extraction Kit. The RNA was analyzed using a nanodrop UV‐visible spectrophotometer (Epoch, Biotek). The cDNA Kit was utilized to reverse transcribe the isolated RNA. The protocol featured an initial incubation at 25°C for 5 min, incubation at 55°C for 30 min, and a final incubation at 85°C for 5 min. GAPDH was employed as a reference gene for this study. The primer sequences for RT‐PCR, synthesized by Sina Colon, are listed in Table 1.

**TABLE 1:** Primer pairs for RT‐PCR.

Gene	Forward primer	Reverse primer	Size (bp)
GAPDH	5′CAATGACCCCTTCATTGACC3′	5′TGGAAGATGGTGATGGGATT3′	141
BAX	5′GCCTCCTCTCCTACTTTG3′	5′CTCAGCCCATCTTCTTCC 3′	106
BCL2	5′TCGCCCTGTGGATGACTGA3′	5′CAGAGACAGCCAGGAGAAATCA3′	138
PI3K	5′TCTTGCTCAGTACAATCCCAAACT3′	5′TCTCCTGATACTGAGAGTGTATTCT3′	94
AKT1	5′TCCTCCTCAAGAATGATGGCA3′	5′GTGCGTTCGATGACAGTGGT3′	183

### 2.12 RT‐PCR

RT‐PCR was performed by SYBR green master mix (Amplicon, Denmark) and Rotor Gene equipment. To sum up, 0.5:1:5 μL of primer, cDNA, and SYBR green master mix were combined. Deionized sterile water was used to achieve the appropriate volume Denaturation was first carried out for 15 s at 95°C. After that, annealing and elongation were completed for 18 and 30 s, respectively, at 62°C and 70°C. Lastly, the relative gene expression data were analyzed using the $2^{-\Delta \Delta C_{t}}$ comparative technique (Salehi et al. 2022).

### 2.13 Statistical Analysis

The MTT results were analyzed using two‐way ANOVA. Duncan's multiple range test was used to calculate significant dissimilarities between data groups in SPSS (v.20). For real‐time data analysis, the REST software was employed. GraphPad Prism 8 was employed to create every graph in this article (*p* < 0.05).

## 3 Results

### 3.1 Nanoparticles Chemically and Physically

**FIGURE 2:**
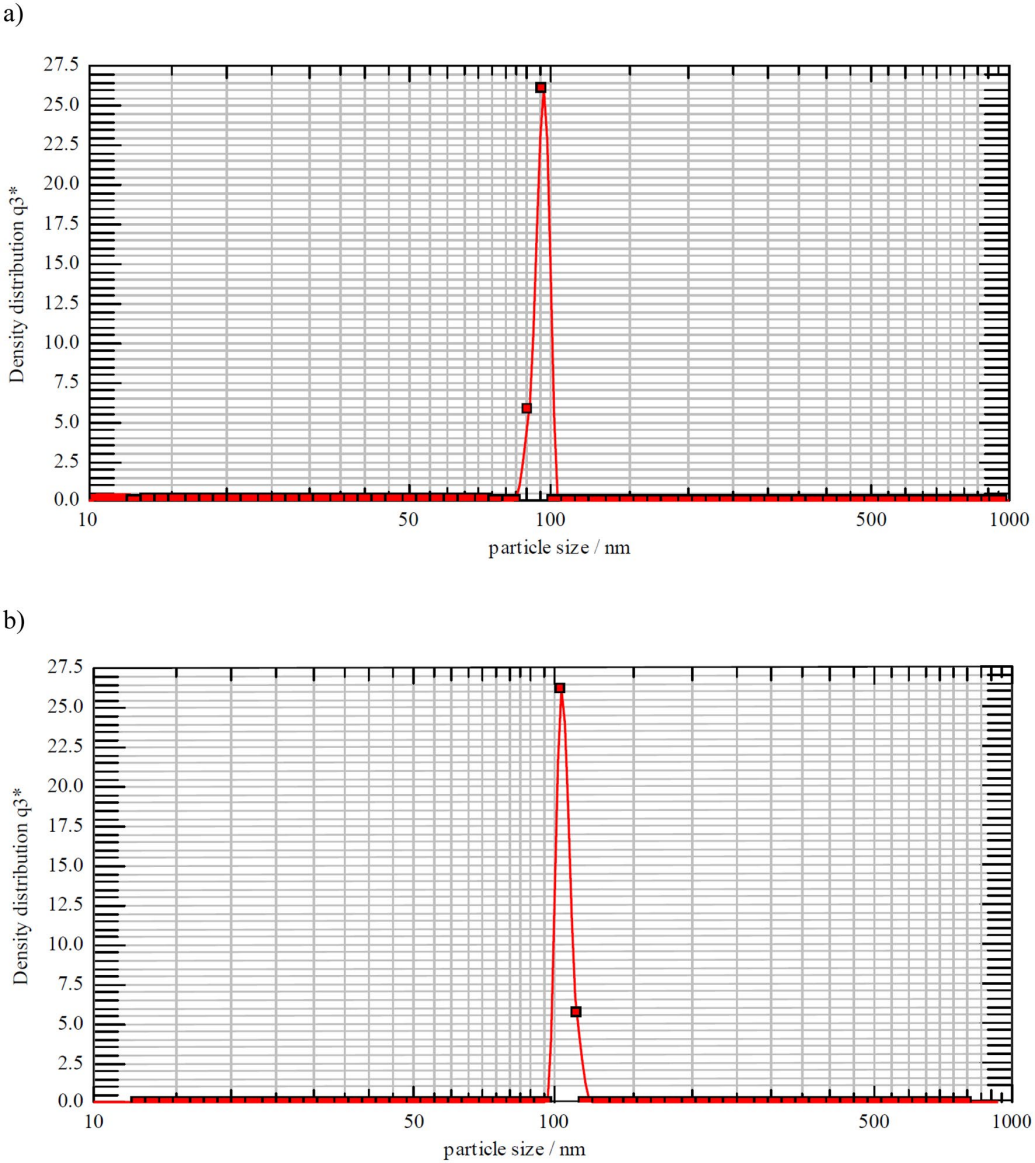
Density‐weighted distribution (a) of the empty liposome and (b) liposome with vitamin D.

**FIGURE 3:**
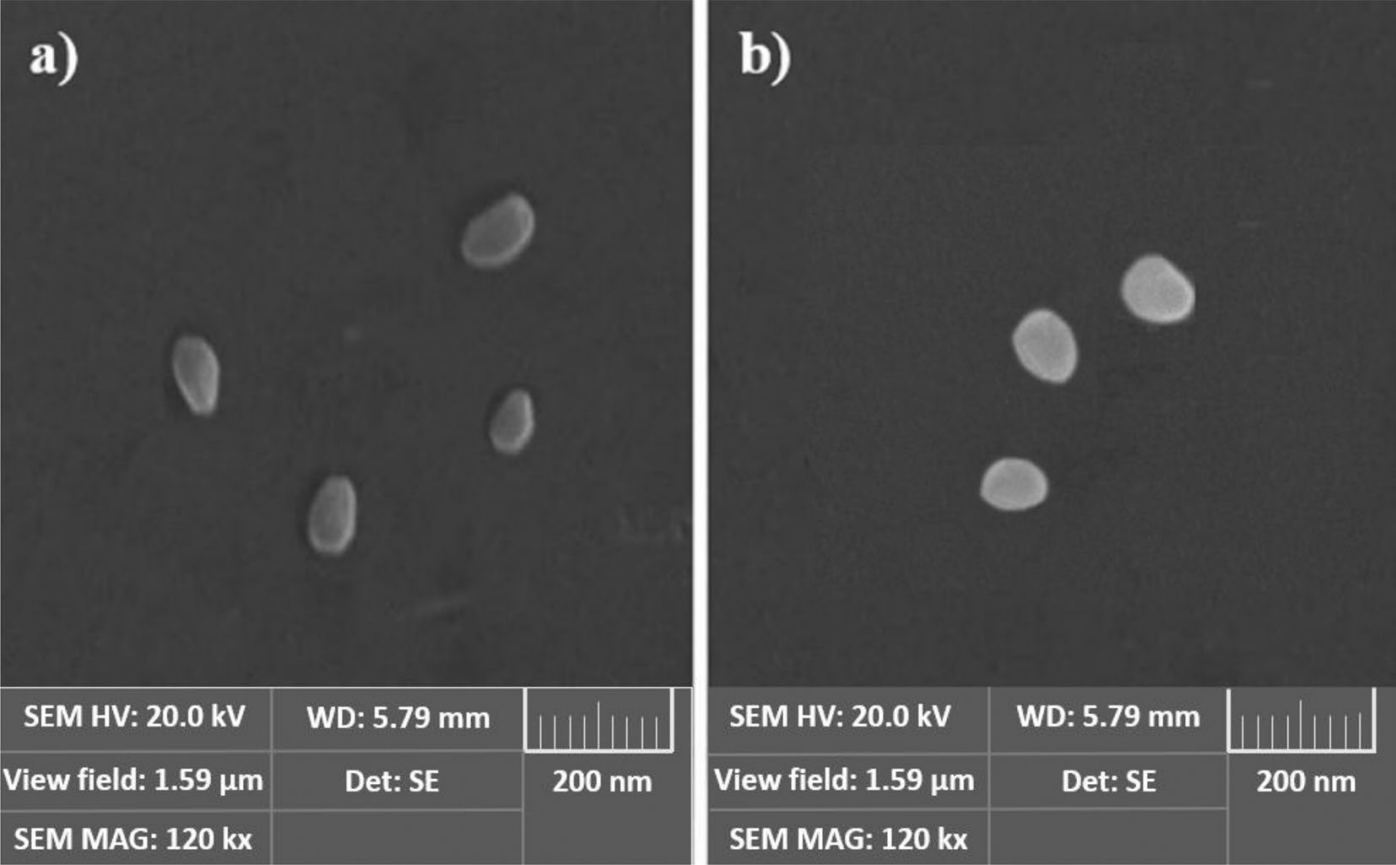
SEM images of (a) empty liposomes, (b) vitamin D liposomes: more spherical and rounder and bigger in size.

Twenty milligram of lipids was needed in the formulation, and the chemical and physical properties of the liposomal structure are listed in Table 2. The nanoliposome distributions are depicted in Figure 2a,b, respectively. SEM was employed to examine the shapes and morphology of empty and loaded liposomes. Figure 3 illustrates that the liposomes are spherical in general and exhibit a similar appearance. Furthermore, the vitamin D‐loaded liposomes displayed a higher level of brilliance in comparison with the empty liposomes. Dynamic light scattering (DLS) analysis indicated that the diameter of vitamin D‐containing liposomes (105.14 nm) was larger than that of empty liposomes (95.29 nm), a finding corroborated by SEM images.

**TABLE 2:** Liposome (Lip) characterization.

Liposomes	Vitamin D (mg)	HSPC (mg)	Cholesterol (mg)	DSPE‐PEG (mg)	Diameter (nm)	PDI	Zeta potential (mV)	Encapsulation efficiency (EE%)
Empty Lip	—	12.76	3.28	3.39	95.29 ± 0.9	0.11 ± 0.05	−3.6 ± 0.26	—
Vit. D Lip	4.5	12.76	3.28	3.39	105.14 ± 1.1	0.12 ± 0.02	−3.9 ± 0.51	90.97 ± 0.08

### 3.2 FTIR Analysis

**FIGURE 4:**
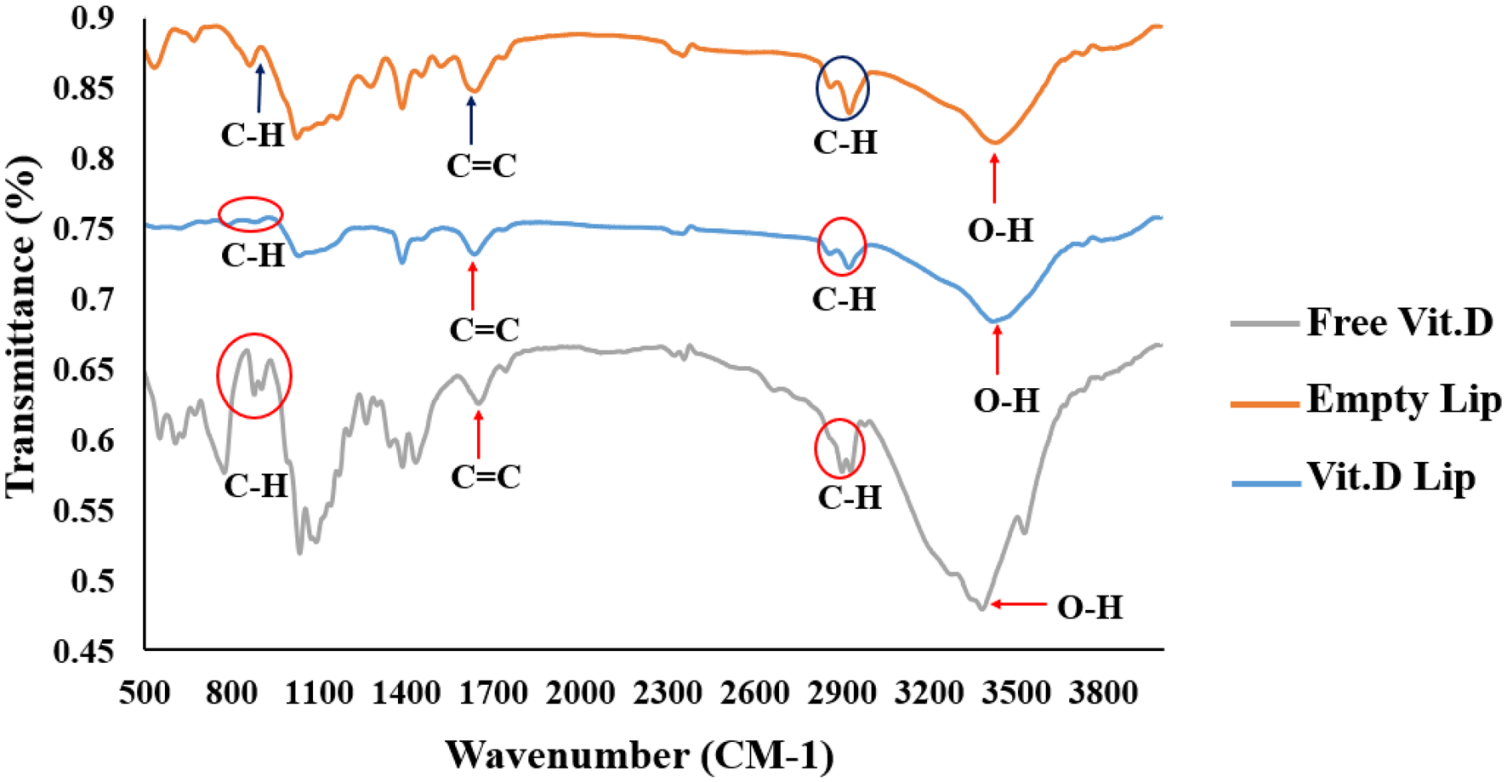
FTIR spectra of free vitamin D, empty liposome, and vitamin D liposome.

FTIR was used to study the interactions among each component through studying changes in band width and frequency of the molecular assembly when vibrating. This analysis aimed to concurrently compare the FTIR of empty liposomes, free and liposomal vitamin D, and also to identify the interactions caused by complex formation. As illustrated, the FTIR results for empty liposomes indicate the presence of aromatic rings within the absorption band range. At 1700 cm^−1^, the band corresponds to C=C stretching vibrations. In the 2400–3000 cm^−1^ range, we can see C–H functional groups. Additionally, consecutive peaks in the 3300–3700 cm^−1^ range refer to the –OH groups. As for the vitamin D spectra, a primary band is seen at 3307 cm^−1^, attributed to –OH group stretching. With encapsulated vitamin D, this peak shifted to a wavenumber of 3405 cm^−1^, indicating the role of hydrogen bonding, mostly in hydroxyl groups. This shift indicates the role of hydrogen bond in the encapsulation of vitamin D. The FTIR spectra of vitamin D‐containing liposomes, empty liposome, and free vitamin D are displayed in Figure 4.

### 3.3 Vitamin D Release Studies

**FIGURE 5:**
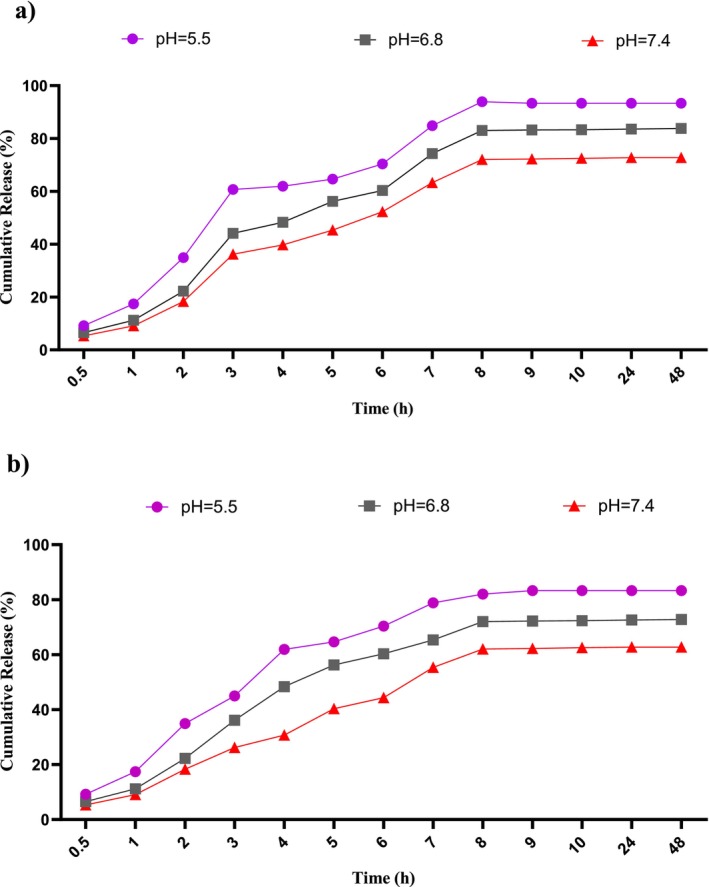
Release percentage of (a) liposomal vitamin D and (b) free vitamin D versus time at different pH values.

The vitamin D release from nanoliposomes was determined at several pH levels (5.5, 6.8, and 7.4) over different time periods. The standard curve was employed to quantify vitamin D release. In 8 h, the volume of vitamin D discharge steadily increased, after which the release rate stabilized. As illustrated in Figure 5a,b, the maximum release happened at pH 5.5, reaching around 93%, in comparison to the releases at pH 6.8 and 7.4. To analyze the release kinetics, a suitable kinetic model was employed, and the results were documented in Table 3. The kinetic modeling analysis indicated that the zero‐order model was the most appropriate for the system at pH 5.5.

**TABLE 3:** Results of investigating the kinetic patterns of vitamin D release in different pH values.

Kinetic model		pH: 5.5	pH: 6.8	pH: 7.4
Zero‐order	*k*_0_	7.99	6.07	8.01
	*r* ^2^	0.998	0.965	0.941
	ss	502.6	1432.5	2325.1
First‐order	*k*_1_	−0.0613	−0.043	−0.086
	*r* ^2^	0.958	0.946	0.973
	ss	49.03	47.11	46.19
Higuchi	*k*_H_	28.06	30.01	30.84
	*r* ^2^	0.961	0.979	0.980
	ss	2342.1	337.04	4376.5
Peppas	*k*_P_	1.51	1.16	1.45
	*r* ^2^	0.983	0.976	0.980
	ss	3.089	1.79	2.07
	*n*	0.523	0.657	0.643
Hixson–Crowell	*k*_C_	−0.129	−0.153	−0.131
	*r* ^2^	0.968	0.978	0.941
	ss	48.11	47.04	49.66

*Note:* Kinetic parameters of release: *k*, rate constant; *n*, release exponent; *r* ^2^, correlation coefficient; ss, sewage sludge.

### 3.4 Cell Viability

**FIGURE 6:**
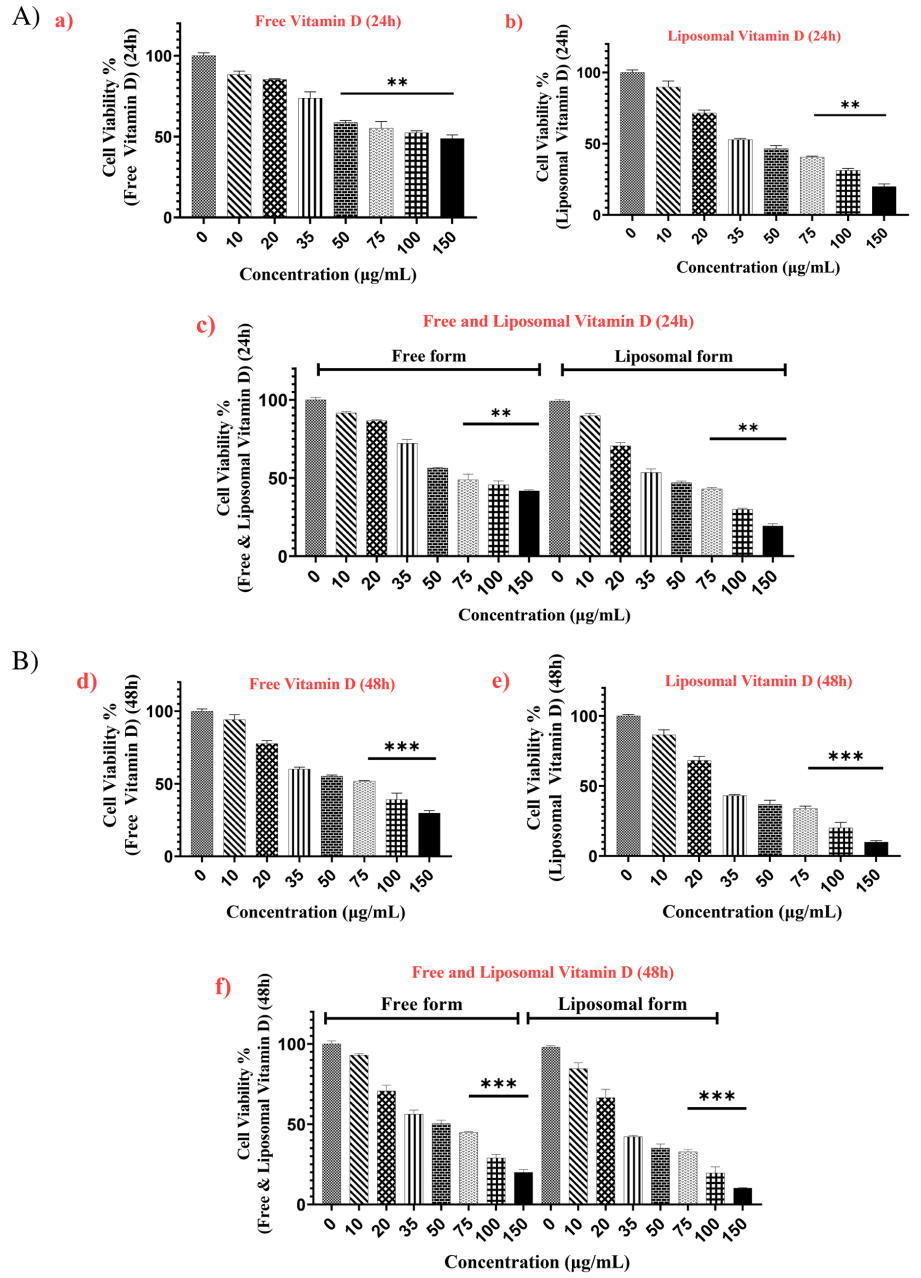
(A) Percentage viability of B16 F10 cells after treated with 0–150 μg/mL doses of (a) free vitamin D for 24 h and (b) liposomal vitamin D for 24 h and (c) comparing the free vitamin D and the liposomal form for 24 h. (B) Percentage viability of B16 F10 cells after treated with 0–150 μg/mL doses of (d) free vitamin D for 48 h and (e) liposomal vitamin D for 48 h and (f) comparing the free vitamin D and the liposomal form for 48 h (*p*‐value < 0.01**/*p*‐value < 0.001***).

The cells were treated with liposomal and free vitamin D at different concentrations. The cell viability was significantly affected by both forms of vitamin D across these concentrations, as shown in Figure 6a,b. The findings validate the toxicity and efficacy of liposomal and free vitamin D at 20–150 μg/mL dosage after 24–48 h of exposure. The IC50 values for cells exposed to vitamin D‐containing liposomes and free vitamin D were determined to be 35.08 and 49.76 μg/mL, respectively, after 24 h, and 28.96 and 41.02 μg/mL, respectively, after 48 h. These IC50 concentrations and the 24–48 h time frame were established for the continuation of the experiments.

### 3.5 Apoptosis

**FIGURE 7:**
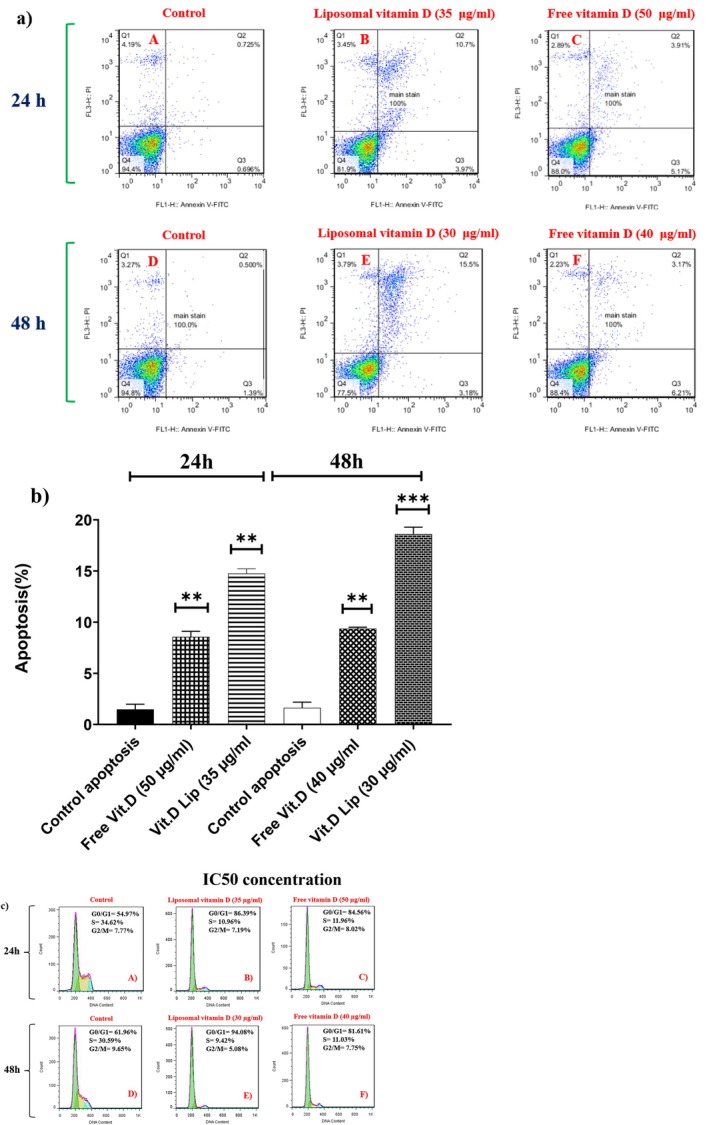
(a) B16 F10 cells were exposed to IC50 doses of two forms of vitamin D. Flow cytometry: Left picture: (A) Control after 24 h, (B) liposomal vitamin D (Vit. D Lip) after 24 h, (C) free vitamin D after 24 h, (D) control after 48 h, (E) liposomal vitamin D after 48 h, (F) free vitamin D after 48 h. (b) Apoptosis after B16 F10 Liposomal vitamin D and Free vitamin D treatments for 24, 48 h. (c) Cell cycle is illustrated according to the IC50 concentrations of compounds. Left picture: (A) Control after 24 h, (B) liposomal vitamin D (Vit. D Lip) after 24 h, (C) free vitamin D after 24 h, (D) control after 48 h, (E) liposomal vitamin D after 48 h, (F) free vitamin D after 48 h (*p*‐value < 0.01**/*p*‐value < 0.001***).

To examine the cellular uptake efficacy of the treatment, apoptotic cell rates were measured based on flow cytometry (Hollville and Martin 2016). Cancer cells were treated with vitamin D‐containing liposomes and free vitamin D for 24–48 h. Notably, after 24 h, the accumulated level of late and early apoptosis for liposomal and free vitamin D at the IC50 dose were 14.67% and 9.08%, respectively. After 48 h, these figures were 9.38% and 18.71% for liposomal and free vitamin D, respectively, as depicted in Figure 7a–c. Moreover, the treatment with vitamin D‐containing liposomes induced a bigger proportion of late apoptosis in cancer cells. In contrast, treatment with free vitamin D led to more extensive induction of early apoptosis. This differential induction of apoptosis stages underscores the enhanced apoptotic efficacy of liposomal vitamin D over its free counterpart.

### 3.6 Cell Cycle Analysis

To determine if liposomal and free vitamin D may affect the advancement of the B16 F10 cell cycle, cell cycle modulation was examined. The results of the exposure to liposomal and free vitamin D (IC50 concentration) showed that after 48 h, the number of cells in the G0/G1 phase increased by 20% and 33%, respectively, in comparison with the control, the number of cells in the S phase decreased by 20% and 22%, and the number of cells in the G2/M phase decreased by 3% and 5%, respectively, in comparison with the control (Figure 7c).

### 3.7 Nanomechanical Characterization

**FIGURE 8:**
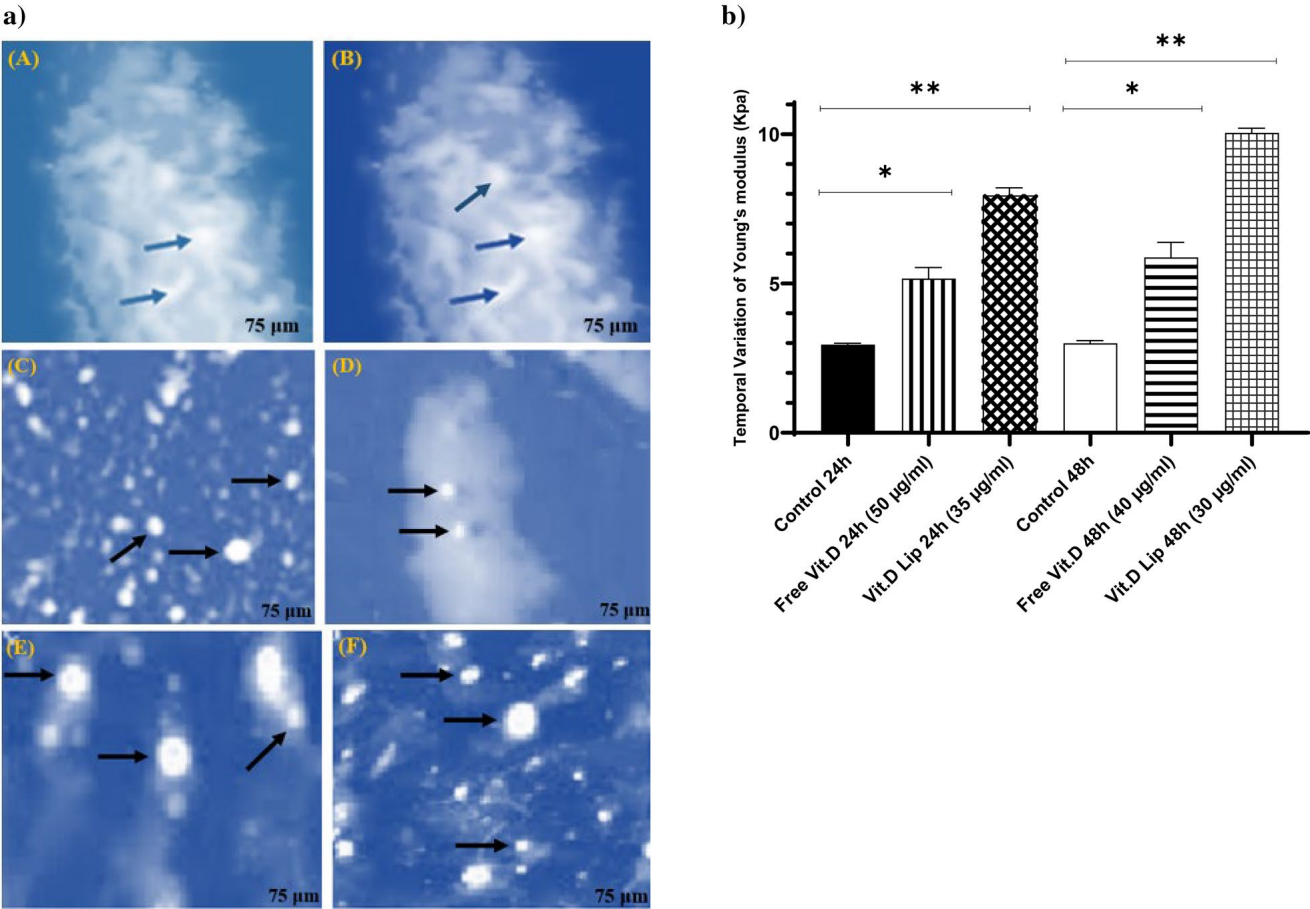
(a) Changes in the cellular membrane ultrastructure of non‐contact mode for the B16 F10 after being treated with free vitamin D and liposomal vitamin D (Vit. D Lip) for 24 and 48 h. (A) Control after 24 h, (B) control after 48 h, (C) free vitamin D after 24 h, (D) free vitamin D after 48 h, (E) liposomal vitamin D after 24 h, (F) liposomal vitamin D after 48 h. The AFM images scale bar is 75 μm. (b) Young's modulus values obtained from B16 F10 cells in various treatments (*p*‐value < 0.05*, *p*‐value < 0.01**, *p*‐value < 0.001***).

The AFM results indicated striations on the topographic surface of the cell. Such structures in AFM topographies are dependent on the subsurface cell's actin cytoskeleton, according to earlier research (Braet et al. 2001; Henderson et al. 1992). Researchers (Poole et al. 2004) noted that flexible surface structures were visible in AFM images. As a result, cells with significant genetic variances can be distinguished by their shared structural traits using the surface AFM technique. AFM was employed to evaluate the two core parts of cancer cells that were in contact (Figure 8a). The filamentous structures were likely actin stress fibers, as actin structures, primarily located beneath the cell membrane, make up the majority of the cytoskeleton (Lekka et al. 1999). Pores and apoptotic bodies were seen in cells treated with vitamin D‐containing liposomes and free vitamin D at the cytoplasmic membrane (Figure 8C–F). Black and blue arrows point to apoptotic bodies and visible pores. At 24–48 h, cells treated with vitamin D‐containing liposomes had greater Young's modulus (Table 4) than control groups. Furthermore, the elastic modulus and adhesive force are directly correlated. With liposomal vitamin D, the cells exhibited the highest cell adhesion force and the lowest mean Z pulling values after 48 h (Table 4). The JPK software analysis verified the alteration of actin filaments in cells exposed to liposomal and free vitamin D. The biggest variations were seen after 48 h of exposing the cells to vitamin D‐containing liposomes (Figure 8b and Table 4).

**TABLE 4:** Changes in mechanical parameters such as Young's modulus, mean Z pulling, and mean adhesion force values of B16 F10 cells.

Compounds	Mean Young's modulus value (kPa) ± SE	Mean Adh. force (pN) ± SE	Mean Z pulling (μm) ± SE	*p*
Control after 24 h	2.97 ± 0.04	2.08 ± 0.32	1.91 ± 0.4	—
Control after 48 h	2.99 ± 0.04	2.17 ± 0.26	1.93 ± 0.5	—
Free vitamin D after 24 h	5.16 ± 1.08	2.34 ± 0.40	3.13 ± 0.08	0.087
Free vitamin D after 48	6.05 ± 0.09	2.69 ± 0.45	1.88 ± 0.6	0.073
Liposomal vitamin D after 24 h	8.03 ± 1.01	4.08 ± 0.07	2.53 ± 1.04	0.069
Liposomal vitamin D after 48 h	10.05 ± 0.8	6.03 ± 0.1	1.63 ± 0.7	0.081

### 3.8 Gene Expression

**FIGURE 9:**
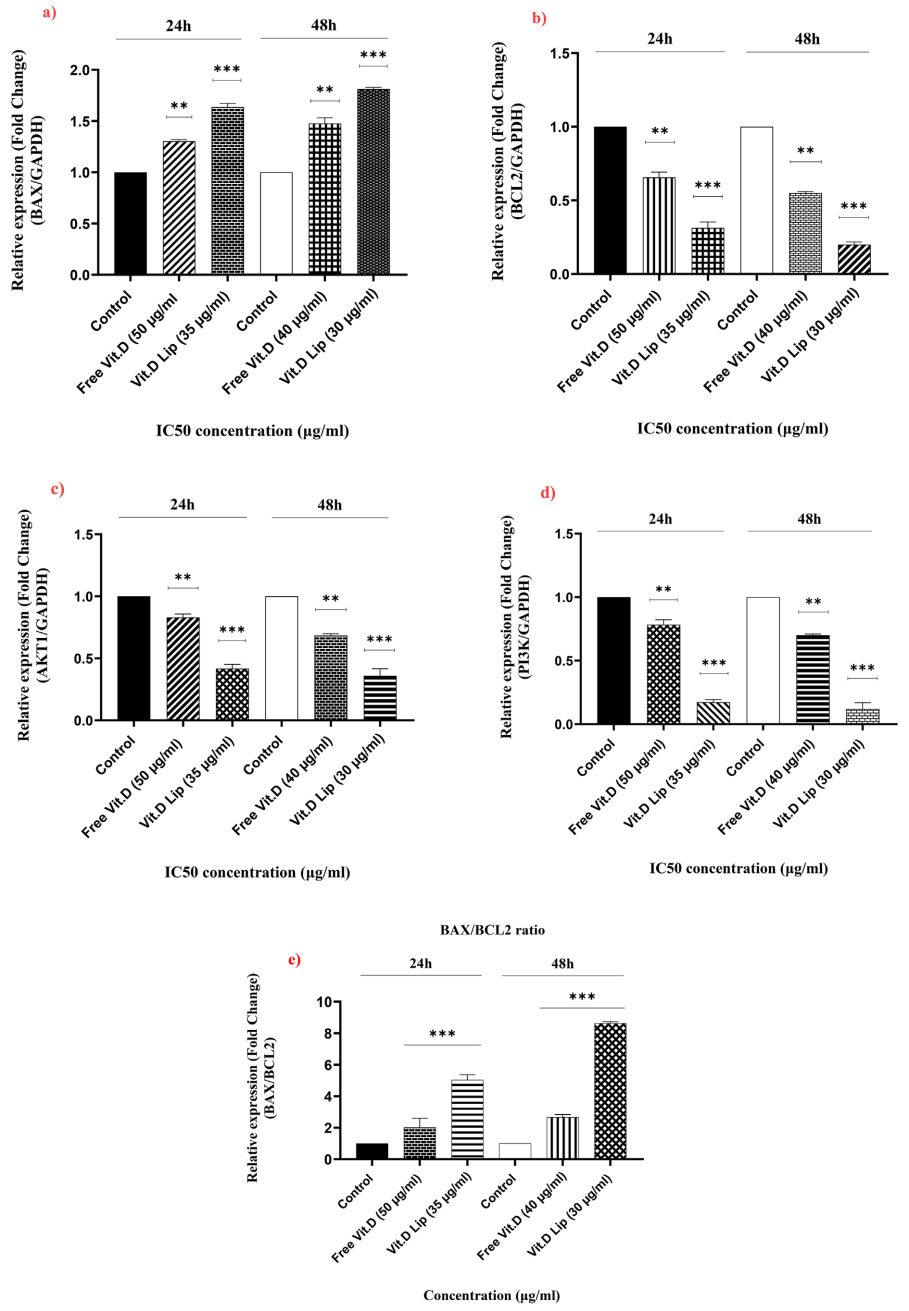
Levels of mRNA expression of BAX, BCL2, AKT1, and PI3K were analyzed using RT‐PCR. The B16 F10 cells were exposed to IC50 doses of free and liposomal vitamin D for 24 and 48 h. In comparison to the cells of control, (a) mRNA expression of BAX increased, whereas (b) mRNA expression of BCL2 decreased. Additionally, (c) mRNA expression levels of AKT1 and (d) PI3K genes decreased in a dose‐independent manner with the treatment of free and liposomal vitamin D. (e) BAX/BCL‐2 ratio (*p*‐value < 0.05*/< 0.01**/< 0.001***).

The apoptotic effect of vitamin D, in its liposomal and free forms on B16 F10 cells was examined. The results, as illustrated in Figure 9a–e, demonstrate a significant increase in the apoptotic BAX gene expression in cells exposed to liposomal and free vitamin D. In contrast, a marked decline in the expression of anti‐apoptotic genes, like BCL2, AKT1, and PI3K was observed.

## 4 Discussion

Using microscopic evaluations, the presence of liposomes carrying vitamin D was examined early in the experiment. The suspension containing liposomes had a light yellow or milky tint. PDI, Particle size, and zeta potential are the key markers to assess the suspended nanoparticles' stability. The size, entrapment efficiency (EE%), zeta potential, and shape of liposomal vitamin D were evaluated. Molaveisi et al. (2021) reported that the nanoliposomes were able of incorporating a substantial volume of vitamin D. The results demonstrated that liposomes efficiency increased with an increase in the dose of HSPC in the structure of liposome, leading to a large number of vesicles. The membrane permeability was reduced with an increase in the concentration of cholesterol in liposome, which in turn lowered EE%. Still, the primary advantage of adding cholesterol was the enhanced stability of the liposome. Furthermore, the attachment of PEG to the surface of the nanoliposomes was observed to enhance the drug's effect by modulating the immune response (Pezeshky et al. 2016; Siepmann et al. 2019). Multiple studies provide strong evidence that zeta potential and liposome stability are positively correlated, with higher zeta potential related to enhanced stability. Table 2 confirms that the liposomal vitamin D has −3.9 mV of zeta potential. Notably, previous research has successfully prepared liposomes of different sizes under a variety of experimental conditions. Makino et al. (1991) and Miatmoko et al. (2019) indicated that liposomes sized in 100 and 150 nm range were generated, which is in good agreement with the liposomes synthesized here. Additionally, the PDI and FTIR results in this project are in agreement with other studies (Mohammadi et al. 2014). As a lipophilic compound, Vitamin D was encapsulated in the liposome. To investigate vitamin D release, three levels of pH were examined. The results showed that with pH = 5.5, vitamin D release increased compared to the other pH levels. The B16 F10 cell line, known for its high metastatic potential, was selected to study vitamin D's efficiency to stop cell growth. The proliferation of B16 F10 cells was successfully suppressed by both forms of vitamin D. In addition, vitamin D‐containing liposomes had a higher suppressive effect on the proliferation of B16 F10 cells. Following treatment with vitamin D‐containing liposomes and free vitamin D, the IC50 values for cancer cells were obtained to be 35.08 and 49.76 μg/mL, respectively, after 24 h. After incubation for 48 h with both forms of vitamin D, the IC50 values were found to be 28.96 and 41.02 μg/mL, respectively. These IC50 results indicate that the proliferation of B16 F10 melanoma cancer cells was significantly suppressed. Previous studies have shown that free vitamin D effectively suppresses B16 F10 cancer cell growth (Ishibashi et al. 2012), consistent with our results. Additionally, our results are consistent with studies on other cell lines, which reported the antiproliferative role of vitamin D (Shariev et al. 2022). Ishibashi et al. (2012) investigated the effects of the concentration of vitamin D on the proliferation and apoptosis of B16 F10 cells, finding significant inhibition effects in the 10–100 μM range (Ishibashi et al. 2012). Furthermore, Evans et al. (1996) studied the effects of vitamin D as a suppressor of cell growth, noting that at 10^−6^ M, it demonstrated the highest impact.

The results of flow cytometry confirmed that vitamin D‐containing liposomes exhibited a higher cytotoxic effect than the free form of vitamin D. Furthermore, the former had a greater rate of late apoptosis. This is consistent with other studies, which showed that the free form of vitamin D triggered AGS cell line apoptosis, with a greater induction of early apoptosis (Alizadeh‐navaei et al. 2019). Similarly, Ishibashi et al. (2012) indicated that melanoma cells (B16 F10) treated with vitamin D had a higher early apoptosis rate. Atomic force microscopy (AFM) is capable of capturing mechanical, ultrastructural, and morphological variations in cells exposed to drugs with no damage to the cells. AFM gives extensive data regarding cells, such as pathology, aging, differentiation, and movement (Tiryaki et al. 2012; Zolghadr et al. 2023). AFM examines Young's modulus, which several prior investigations have suggested decreases with an increase in malignancy. Variations in the organization of actin filaments in malignant cells result in lower adhesion potential and increased pliability (Pi et al. 2016), findings that align with the present study. The results indicated that liposomal and free vitamin D significantly impacted gene expression in the B16 F10 cell line. Both forms of vitamin D enhanced the BAX gene expression along with attenuating the expression of the BCL2 gene at the same time. Kizildag et al. showed that using vitamin D on the K562 cell line decreased mRNA expression of the BCL2 and BCL‐XL genes, while increasing the expression of apoptosis‐inducing genes (P21 and BAX).

Additionally, this research found that liposomal and free vitamin D impacted and hindered the PI3K/AKT/mTOR and Ras/ERK pathways in the B16 F10 cells by lowering PIK3R1 and AKT1 gene expression. The findings are consistent with previous research.

## 5 Conclusions

To encapsulate vitamin D in the liposomes, the thin layer hydration was used. According to the results, the liposomes released their maximum amount of vitamin D at pH = 5.5, which is often the ideal pH for the growth of cancer cells. Vitamin D release from the nanoliposomes was studied by using different models of drug release. The zero‐order model showed that the maximum coefficient of correlation was achieved with pH = 5.5. The two forms of vitamin D were able to induce apoptosis in melanoma cells. Still, vitamin D‐containing liposomes proved more effective than the free form. AFM analyses showed that with vitamin D‐containing liposomes, cancer cells had greater cell–cell adhesion force and elastic modulus. Furthermore, exposed to liposomal vitamin D, there was a higher rise in the BAX gene expression. Conversely, with liposomal vitamin D, the cells exhibited a more marked decline in the AKT1, BCL2, and PI3K gene expression. Still, liposomal vitamin D demonstrates drawbacks like high production costs and low solubility. In addition, the phospholipids of the liposome might be oxidized and, in some cases, encapsulated vitamin D might leak.

## Author Contributions

**Azita Bahrami:** data curation (equal), formal analysis (equal), investigation (equal), methodology (equal), software (equal), validation (equal), writing – original draft (equal), writing – review and editing (equal). **Alireza Farasat:** conceptualization (equal), methodology (equal). **Nematollah Gheibi:** funding acquisition (equal), project administration (equal). **Leila Zolghadr:** interpretation of data and assisted in analyzing the results. **Yalda Sabaghi:** assisted in conducting laboratory experiments.

## Ethics Statement

The authors have nothing to report.

## Consent

The authors have nothing to report.

## Conflicts of Interest

The authors declare no conflicts of interest.

## Data Availability

The data that support the findings in the present research are accessible through the corresponding author on reasonable demand.
